# Interpreting prognostic asymmetry in heart failure–chronic obstructive pulmonary disease overlap

**DOI:** 10.1093/eschf/xvag222

**Published:** 2026-08-24

**Authors:** Huijuan Cheng, Zhe Zhang, Feng Lu

**Affiliations:** Department of Respiratory Medicine, The Second Affiliated Hospital of Fujian University of Traditional Chinese Medicine, No. 282 Wusi Road, Gulou District, Fuzhou, Fujian Province 350003, China; The First Clinical Medical College, Fujian University of Traditional Chinese Medicine, Fuzhou, Fujian Province 350122, China; The First Clinical Medical College, Fujian University of Traditional Chinese Medicine, Fuzhou, Fujian Province 350122, China; Department of Respiratory Medicine, The Second Affiliated Hospital of Fujian University of Traditional Chinese Medicine, No. 282 Wusi Road, Gulou District, Fuzhou, Fujian Province 350003, China

**Keywords:** Chronic obstructive pulmonary disease, Heart failure, Dyspnoea, Mortality, Selection bias

Dear Editor,

We read with interest the study by Baudry *et al.*,^1^ which examined the prognostic interplay between acute heart failure (HF) and chronic obstructive pulmonary disease among 5131 patients hospitalized for acute dyspnoea. The finding that concomitant chronic obstructive pulmonary disease was associated with lower in-hospital mortality but higher post-discharge mortality in the primary HF cohort, whereas concomitant HF was associated with substantially higher long-term mortality in the primary chronic obstructive pulmonary disease cohort, is clinically important. However, the interpretation of this pattern as an asymmetric prognostic effect warrants further consideration.

Relevant external evidence supports the adverse long-term association of chronic obstructive pulmonary disease within HF. A meta-analysis of 11 studies involving 18 602 patients with heart failure with preserved ejection fraction (HFpEF) found that chronic obstructive pulmonary disease was associated with higher mortality (risk ratio [RR] 1.62, 95% confidence interval [CI] 1.34–1.95), including higher post-discharge mortality (RR 2.57, 95% CI 1.34–4.93).^2^ However, that analysis evaluated chronic obstructive pulmonary disease as a prognostic factor within HFpEF and did not examine the reciprocal prognostic impact of HF within chronic obstructive pulmonary disease. It therefore supports the prognostic relevance of chronic obstructive pulmonary disease comorbidity but does not itself establish the bidirectional prognostic asymmetry proposed in PARADISE.

The central conclusion that HF carries a greater prognostic impact in patients with chronic obstructive pulmonary disease than chronic obstructive pulmonary disease does in patients with HF is based on adjusted hazard ratios of 1.48 and 1.22, respectively. These estimates were obtained from separate models with different index populations, reference groups, and baseline hazards. A numerical difference between two effect estimates does not itself establish statistically significant heterogeneity. Formal assessment of interaction is required when the scientific question concerns whether an association differs across strata.^3^ A combined model, including primary diagnosis, overlap status, and their interaction, or an explicit ratio of hazard ratios with a CI, would therefore help determine whether the reported ‘asymmetry’ exceeds what could arise from sampling variation.

A related issue concerns selection into the post-discharge risk set. Five-year mortality was modelled only among patients who survived the index hospitalization. Yet in-hospital mortality differed materially by overlap status: 8.5% vs 11.7% in the primary HF cohort and 7.4% vs 3.4% in the primary chronic obstructive pulmonary disease cohort. Conditioning long-term analyses on discharge alive can therefore select different subsets of patients in each group. In the HF cohort, lower in-hospital mortality among patients with chronic obstructive pulmonary disease allows more high-risk individuals to enter the post-discharge analysis; in the chronic obstructive pulmonary disease cohort, higher early mortality among those with HF may remove some of the most vulnerable patients before follow-up begins. Such conditioning can induce selection or collider bias and may alter the magnitude of post-discharge hazard ratios.^4^ A multi-state framework beginning at hospital admission, with transitions from hospitalization to in-hospital death or discharge and subsequently from discharge to death, could integrate the acute and chronic phases without conditioning the long-term comparison on survival to discharge.

The use of a primary discharge diagnosis as the stratifying variable deserves similar caution. Primary diagnosis was assigned at the end of hospitalization, while the alternate condition was identified from history or a secondary discharge diagnosis. In acute dyspnoea, HF and chronic obstructive pulmonary disease frequently overlap diagnostically, and prospective cardiopulmonary evaluation has shown that both conditions may remain unrecognized even in specialized clinics.^5^ Because diagnostic prioritization can be influenced by presenting physiology, testing, treatment response, and disease severity, stratification by the final ‘primary’ diagnosis may partly reflect the diagnostic pathway rather than a fixed baseline phenotype. Sensitivity analyses based on a four-group classification defined independently of diagnostic primacy, together with admission physiological variables already available in the cohort, could help test the robustness of the observed pattern.

These considerations do not diminish the clinical relevance of the PARADISE cohort. Rather, formal interaction testing and analyses that account for the transition from hospitalization to discharge would clarify whether the reported prognostic asymmetry represents a true difference in HF–chronic obstructive pulmonary disease interplay or is partly shaped by diagnostic and survival selection.

## Declarations

### Data Availability

No new data were generated or analysed in support of this manuscript.
